# The uptake of key elements of sexual and reproductive health services and its predictors among rural adolescents in Southern Ethiopia, 2020: application of a Poisson regression analysis

**DOI:** 10.1186/s12978-023-01562-7

**Published:** 2023-01-12

**Authors:** Aklilu Habte, Samuel Dessu

**Affiliations:** 1 School of Public Health, College of Medicine and Health Sciences, Wachemo University, Hosanna, Ethiopia; 2 Department of Public Health, College of Medicine and Health Sciences, Wolkite University, Wolkite, Ethiopia

**Keywords:** Adolescents, Determinants, Sexual and reproductive health, Ethiopia

## Abstract

**Background:**

Although 25% of the Ethiopian population is young, Sexual and Reproductive Health (SRH) Services have not been adequately researched and intervened, leaving adolescents with many reproductive health challenges. Assessment of the uptake of each element of SRH service and its determinants among those age groups is essential to improving service uptake and reducing the burden of illness and disability in adolescents. Thus, this study aimed at assessing the uptake of key elements of SRH services and its determinants among adolescents residing in rural districts of Guraghe zone, Southern Ethiopia.

**Methods:**

A community-based cross-sectional study was carried out from May 1 to 30, 2020, and a multi-stage sampling technique was employed to randomly select 1028 adolescents. The data were collected by using a pre-tested interviewer-administered questionnaire. The data were coded and entered into Epi-Data version 3.1 and exported into SPSS version 23 for analysis. Independent t-tests and analysis of variance (ANOVA) were run to determine whether there were statistically significant differences in the mean number of SRH services used across each categorical variable. A multivariable generalized linear regression (GLM) model with a Poisson link was used to determine the effect of each variable on the mean number of SRH services used. Adjusted odds ratios with their corresponding 95% confidence interval were used to declare the statistical significance of the independent variables.

**Results:**

The study included 1,009 adolescents, yielding a response rate of 98.1%. The use of the SRH service was assessed using eight elements, and the mean (± SD) score of service uptake was 4.05 (± 1.94), with only 6.8% of adolescents receiving all key elements. Comprehensive sexuality education (55.1%) and voluntary HIV/AIDS counseling and testing (51.0%) were the commonest service items used by adolescents, while the provision of contraceptives was the lowest service item received (25.9%). Educational level (AOR: 1.28, 95% CI: 1.03–1.56), having a parental discussion (AOR: 1.31, 95% CI: 1.13–1.51), lack of youth clubs (AOR: 0.71, 95% CI: 0.66–0.87), and knowledge on SRH issues (AOR: 0.79, 95% CI: 0.73–0.85) were identified as significant predictors of the uptake of key elements of SRH services.

**Conclusion:**

The overall uptake of SRH services was found to be low in the study area. Schools should be an excellent means of educating adolescents to increase their knowledge of key elements of SRH services. Furthermore, stakeholders must work together to improve the culture of parental discussion with adolescents and establish and strengthen youth clubs, as measures for encouraging the use of SRH services.

## Plain language summary

Progress towards universal health coverage requires keeping adolescents healthy, as adolescence represents a significant opportunity for successful prevention and health promotion with life-course implications. Sexual and reproductive health (SRH) service is well described as the combination of techniques, procedures, and services that prevent and respond to sexual health snags by ensuring sexual health and well-being. While most adolescent health problems are avertible or fixable, adolescents face many hurdles in accessing health care and information, especially those who are in rural corners. Although 25% of the Ethiopian population is young, Sexual and Reproductive Health (SRH) Services have not been adequately researched and intervened, leaving adolescents with many reproductive health challenges. Assessment of the uptake of each element of SRH service and its determinants among those age groups is essential to improving service uptake and reducing the burden of illness and disability in adolescents. Thus, this study aimed at assessing the uptake of key elements of SRH services and its determinants among adolescents residing in rural districts of Guraghe zone, Southern Ethiopia. A multi-stage method of sampling was employed, and adolescents have been randomly chosen and participated in the study. Pre-tested structured questionnaires were used for data collection. Statistical significance between dependent and independent variables was checked by using bivariate analysis across ANOVA and independent t-tests. To see the weight of each explanatory variable on SRH service utilization, a generalized linear regression model (GLM) with a Poisson link was done. The overall uptake of SRH services was found to be low in the study area. Comprehensive sexuality education (55.1%) and voluntary HIV/AIDS counseling and testing (51.0%) were the commonest service items used by adolescents, while the provision of contraceptives was the lowest service item received (25.9%). Educational level, having a parental discussion, lack of youth clubs, and knowledge of SRH issues were identified as significant predictors of the uptake of key elements of SRH services. Schools should be an excellent means of educating adolescents to increase their knowledge of key elements of SRH services. Furthermore, stakeholders must work together to improve the culture of parental discussion with adolescents and establish and strengthen youth clubs, as measures for encouraging the use of SRH services.

## Background

Adolescents are people between the ages of 10 and 19, According to the World Health Organisation (WHO) [1]. Adolescence is a decisive phase of human growth with rapid physical, psychosocial, intellectual, and emotional development and erotic and reproductive maturation [1, 2]. The Sexual and Reproductive Health (SRH) service is well described as the combination of techniques, procedures, and services that prevent and address sexual health problems by ensuring sexual health and well-being [3, 4]. Adolescent SRH services include comprehensive sexuality education, promotion of safe sexual behaviors, counseling and provision of modern contraception, HIV and other STI testing and management, safe abortion and post-abortion care, parental and/or peer-to-peer discussion on SRH issues, and effective referral linkage [5–7].

There are approximately 1.2 billion adolescents globally. Adolescents constitute up to a quarter of the population in some countries, and the number of adolescents is expected to increase by 2050, particularly in low- and middle-income countries (LMICs), where access to health and social services, jobs, and livelihoods appears to be under pressure [8, 9]. In sub-Saharan Africa, adolescents represent 23 percent of the region's population[9]. About 25 percent of Ethiopia's total population is covered by a cohort of young people [6, 10].

Despite international initiatives emphasizing adolescents' rights to SHR services, neither the providers of these services nor the systems in which they operate are equipped to meet the needs of adolescents in developing countries [11–13]. Most countries' health systems and programs are designed primarily for adults, with less emphasis placed on SRH services for adolescents [12]. As a result, the progress in mortality among adolescents has been delayed [13, 14]. There are over 1.2 million adolescent deaths worldwide every year [8, 13]. Adolescent pregnancy, often related to child marriage or reduced participation in education, has been associated with low maternal health outcomes [15, 16]. Every year, close to 16 million adolescent girls gives birth, with the majority of these births occurring in early marriages, and 90% in developing countries [15].

The adoption of sexual and reproductive health (SRH) services among adolescents have not been adequately researched or implemented, despite the fact that 23% of the SSA population was adolescent [16]. Up to 68% of adolescents in this region have an unmet need for contraception [17]. In the country Ethiopia, where about 25% of the population is young, adolescents face numerous sexual and reproductive health (SRH) challenges such as unplanned pregnancies and adolescent pregnancies, unsafe abortion, sexually transmitted infections (STIs), including HIV/AIDS and intimate violence [6, 10].

According to the 2019 Mini Ethiopian Demographic and Health Survey (2019 mini EDHS), contraceptive use in currently married women aged 15–19 was only 36.5%, with 27.5%, 5.9%, and 0.0% injectables, implants, and IUDs respectively[18]. At 75% and 80% respectively, the proportion of adolescents who never tested for HIV is highest among females and males aged 15 to 19 years [19]. Studies conducted elsewhere in Ethiopia showed that access to SRH services is generally insufficient [20–23].

Adolescents must be kept healthy to progress toward universal health coverage, as adolescence represents a critical opportunity for the successful future of the nations [12]. For every dollar spent on adolescent health, the return to health, society, and the economy is approximately ten times greater [24]. As a result, governments must reach out to adolescents in their day-to-day activities with a set of high-quality, well-organized, and well-integrated interventions, which may be provided through health services to meet the needs of adolescents [12]. The Federal Ministry of Health (FMOH) of Ethiopia has introduced various intervention strategies to enhance national SRH service uptake for adolescents and young people to overcome SRH problems [6, 10]. Despite those interventions, there is still a significant gap in access to SRH services for adolescents and young adults in Ethiopia, especially in rural parts [6].

It is critical to assess the level and determinants of the use of key SRH services in this age group in order to increase uptake and reduce the burden of preventable morbidities and mortalities. Furthermore, understanding and appreciating the pattern of SRH service utilization among adolescents will aid in future planning for better service delivery by increasing knowledge and skills and providing a safe and supportive environment [11]. Most studies on this issue have been limited to urban areas and have also failed to address the level of uptake of each service package item. Thus, this study aimed at assessing the uptake of key elements of SRH services and its determinants among rural adolescents in Southern Ethiopia.

## Methods

### Study area, period and design

A community-based cross-sectional study was conducted in the Gurage Zone, central Ethiopia from May 1 to 30, 2020. The area is located 158 km from Addis Ababa (Ethiopian capital) and 337 km from Hawassa (SNNPR capital). The adolescent population (15–19 yrs) accounted for 13.6% of the total population. The area is divided into 11 administrative districts encompassing 174 rural kebeles *(the smallest administrative unit next to one district of the Ethiopian government*). There were 128 health facilities in total, which included 74 health centers, 5 hospitals, 168 health posts, and 30 private clinics.

### Population

All adolescents in rural districts of the Guraghe zone were the source population, and all adolescents in the selected districts in the zone during the study period were the study population. The study excluded participants who have lived in the study area for less than six months and adolescents who were seriously ill at the time of data collection.

### Sample size determination and sampling techniques

Using the single population proportion formula, the sample size was determined by considering the following parameters: the proportion of 41.2% (i.e. adolescents who received SRH services and taken from a study conducted in northwest Ethiopia [20], a 95% confidence level, 5% margin of error, 10% non-response, and 2.5 design effect. This resulted in a sample size of 1028 for the study. A multi-stage sampling technique was used to select study participants. Five districts have been randomly selected in 11 rural districts, namely: Ezha (with 24 kebeles), Cheha (with 12 kebeles), MihurAkilil (with 12 kebeles), Meskan (with 11 kebeles), and Soddo (11kebles). Then 28 kebeles were randomly selected from among the five aforementioned districts. The sample size was proportionately distributed among each eligible kebele. With the help of community health workers (CHWs), households with eligible adolescent/s were coded and a sampling frame was formed. It was convenient to have access to every participant in the study through simple random sampling. In the selected Kebele, those eligible participants who were not available during data collection were re-visited three times. When more than one adolescent was availed in the selected household, a lottery method was used to select one of them.

### Data collection tools, methods, and personnel

Pre-tested structured questionnaires were prepared in the context of previous studies in the fields of interest [10, 12, 20–22]. The questionnaire had multiple segments: socio-demographic and economic characteristics, access to SRH services, knowledge of SRH issues, lifestyle and sexual activity of respondents, and use of SRH services. The data were collected through an interviewer-administered questionnaire by 14 trained diploma nurses with data collection experience, under the supervision of six public health officers.

### Data quality management

To ensure accuracy, the data collection tool was translated from English to Amharic by experts in that language. A pre-test was conducted on 5% of the sample size (52 adolescents) who lived outside of the study area, and changes were made based on the results of the pre-test to improve responses to the questionnaires. The reliability of the questionnaires was assessed by SPSS using the reliability index for practical questions (Cronbach’s alpha), which was 0.77. continuous supervision was done throughout the data collection period. The data collected were checked for completeness and inconsistencies before analysis.

### Data analysis

The data were entered into EpiData version 3.1 and exported to SPSS version 23 for analysis. Using descriptive statistical analysis, the frequency, percent, and mean of explanatory and response variables were computed. By using bivariate analysis through ANOVA and independent t-tests, statistical significance was tested between dependent and independent variables. In bivariate statistical analysis (ANOVA and Independent t-test), variables with p-value ≤ 0.05 was considered as candidate for multivariable statistical analysis (generalized linear model with Poisson regression). The determinants of the use of SRH services have been established using the Generalized Linear Model (GLM) technique. The Poisson regression analysis was carried out because our outcome variable of interest was calculated in terms of counting variables. It satisfies the equidispersion assumption as it tests the Poisson regression model assumption[25]. Finally, odds ratios and 95% confidence intervals have been calculated for each independent variable.

## Definition and operationalization of variables of the study

### Dependent variable

Sexual and reproductive health (SRH): is a constellation of services comprising: consultation and provision of modern contraceptives; counseling and provision of safe abortion care; prevention and care of STIs; prevention and care for HIV/AIDS; provision of comprehensive sexuality education(CSE); engagement in peer-to-peer education in their village or school on SRH issues; prevention and care of violence against women and girl(VAWG); and counseling on prevention of harmful traditional practice(HTPs) [4–7, 11, 22]. For each service element, response categories have been established as ‘YES = 1' and ‘NO = 0'. A composite index was developed that summarizes the use of SRH services by adding the above service elements. The variable had the lowest value of zero, indicating that no SRH services were used, and the highest value of eight, indicating that all SRH service elements were used.

Adolescent: In this study, adolescents are boys and girls aged 15–19 [21, 26, 27].

Comprehensive sexuality education (CSE): When adolescents were comprehensively offered at least one element of the CSE within the last 12 months from health workers working in any of the service delivery points; awareness creation on reproduction, having information about a positive and respectful approach to sexual relationships, prevention of SRH problems, rights-based approach to SRH services, prevention of violence against women and girls, mental health, and approaches to life skills to enhance their sexual, physical, and emotional wellbeing [4, 21, 28, 29].

### Independent variables

Discussion on SRH issues: Adolescents who have discussed at least two SRH issues over the past 12 months (condom use, STIs/HIV/AIDS, abstinence, unwanted pregnancy, contraception) with health care providers, peers, sexual partners, and/or parents [21].

Modern contraceptive service utilization: Adolescents who, over the past 12 months, have used one of the modern methods of contraception (oral contraceptives, condoms (males and females), injectables, implants, intrauterine devices, emergency contraceptive pills and spermicide agents) [21, 26].

Accessibility to SRH service: This applies to the perceived distance respondents traveled to reach SRH service delivery points and/or the amount of time they spend. Adolescents residing within a 1.6 km radius of the nearest HRS service center and/or reaching these service delivery points within a 30-min walk were classified as having good geographical access [21].

Substance use: Use of addictive substances such as alcohol, Khat or cigarettes at a frequency of; more repeated than daily, daily, weekly or monthly during the 12 months prior to the study [21].

Reproductive health service knowledge: Adolescents were asked twelve questions about their perceptions of SRH-related issues. An index was created to summarize the level of knowledge, and those who scored higher than the mean were classified as knowledgeable, while those who scored lower were classified as not knowledgeable [21, 22].

Availability of Youth clubs: Accessibility of places/rooms where young people can meet and gather SRH information, SRH services such as contraceptives, physical activities, social support, and peer-to-peer discussion, with the aid of trained workers and volunteers to protect adolescents from negative events, anti-social behavior, crime, drug, and alcohol abuse that are a problem in this community[30–32].

## Results

### Background characteristics of respondents

One thousand-nine rural adolescents took part in the study, resulting in a response rate of 98.1%. The average age of adolescents was 17.07 ± 1.4 years and females accounted for more than half (57.1%). The largest proportion of adolescents (95.9%) was single and more than half (51.8%) went to secondary school. Guraghe constituted the majority (90.8%) of the ethnicity and, according to religion, more than half (54.9%) were Orthodox Christians. The average sizes of families were 5.15 ± 1.55. Concerning ease of geographic access, fewer than half (43.3%) of participants had access to these service delivery points within a 30-min walk of their homes. Health centers (60.2%) and private clinics (35.7%) were among the commonly accessed service delivery points. Other service delivery points that adolescents had access to were private pharmacies (32.4%), hospitals (24.5%), and health posts (13.8%). More than four in ten (41.7%) said there were youth clubs (YCs) nearby (Table 1).

**Table 1:** Background characteristics of rural adolescents in Guraghe zone, Southern Ethiopia, (n = 1009)

Respondent’s characteristics	Frequency	%
Age		
15–16	413	40.9
17–19	596	59.1
Sex		
Male	426	42.2
Female	583	57.3
Marital status		
Ever Married	41	4.1
Unmarried	968	95.9
Religion		
Orthodox	554	54.9
Muslim	380	37.7
Protestant	47	4.7
Catholic	28	2.8
Ethnicity		
Guraghe	917	90.9
Amhara	66	6.5
Wolaita	15	1.5
Hadiya	11	1.1
Current enrolment at school		
Yes	916	90.8
No	93	9.2
Educational status		
No formal education	81	8.1
Primary	405	40.1
Secondary	523	51.8
Current living arrangement		
With both parents	864	85.6
With mother only	81	8.0
With father only	40	4.0
With husband or wife	24	2.4
Mother’s education level (n = 969)		
No Formal education	531	54.8
Primary	300	31.0
Secondary	99	10.2
Diploma and above	39	4.0
Father’s education		
No formal education	387	41.7
Primary	321	34.6
Secondary	160	17.2
Diploma and above	60	6.5
Family size		
≤ 5	543	53.8
> 5	466	46.2
Geographical accessibility to nearby SRH service delivery points		
Accessible	437	43.3
Not accessible	572	56.7
Availability of Youth clubs nearby		
Yes	421	41.7
No	588	58.3
Participation in Youth clubs(n = 425)		
Yes	182	42.8
No	243	57.2

### Respondents’ attributes related to sexuality and reproductive health

Of the participants as a whole, 267 (26.5%) had sexual partner/s, of which 62.5% had one sexual partner (mean = 1.49, SD ± 0.67). Over the past 12 months, 443 (43.9%) study participants reported that they had had a parental discussion on SRH issues. The main topics covered during the discussion were abstinence (60.9%), preventing unwanted pregnancies (77.6%), preventing STIs/HIV/AIDS (62.3%), and contraception (28.4%). In terms of substance use in the previous 12 months, 119 (11.8%), 146 (14.5%), and 103 (10.2%) of respondents used alcohol, Khat, and cigarettes, respectively (Table 2).

**Table 2:** Individual attributes related to sexuality and reproductive health among rural adolescents of Guraghe zone, southern Ethiopia, 2020 (n = 1009)

Respondent characteristics	Frequency	%
Ever had sexual partner/s (n = 1009)		
Yes	267	26.5
No	742	73.5
Number of sexual partners (n = 267)		
One	167	62.5
More than one	100	37.5
Ever had of sexual intercourse (n = 267)		
Yes	217	81.3
No	50	18.7
Use contraceptives during their first sexual intercourse (n = 267)		
Yes	165	61.7
No	102	38.3
Had sexual intercourse within the last 12 months (n = 267)		
Yes	187	70.0
No	80	30.0
Frequency of sexual intercourse (n = 187)		
Once	25	13.4
> Once with the same partner	100	53.5
> Once with a different partner	62	33.1
Perceived risk towards HIV/AIDS nfection		
Yes	152	15.1
No	857	84.9
Ever had a parental discussion on SRH issues		
Yes	443	43.9
No	566	56.1
Counseled and Provided with modern contraception		
Yes	261	25.9
No	748	74.1
Contraceptive utilization by method mix (n = 261)		
Oral contraceptives	134	51.3
Injectables	72	27.6
Condom	223	85.4
Implants	52	19.9
IUD	13	4.9
Alcohol consumption pattern		
Ever users	164	16.2
Drunk Within the last 1 yr	119	11.8
Within the last 3 months	64	6.3
Frequency of alcohol consumption (n = 164)		
Almost every day	4	2.4
At least once a week	14	8.5
At least once a month	52	31.7
At least once a year	58	35.5
Ceased currently*	36	21.9
‘Khat’ chewing pattern		
Ever chewer	246	24.4
Within the last 12 months	146	14.5
Within the last 3 months	113	11.2
Frequency khat chewing (n = 246)		
Almost every day	9	3.6
At least once a week	52	21.2
At least once a month	88	35.7
At least once a year	71	28.9
Ceased currently*	26	10.6
Patterns of Cigarette smoking		
Ever smoke	113	11.2
Within the last 12 months	103	10.2
Within the last 3 months	48	4.7
Frequency smoking (n = 113)		
Almost every day	3	2.6
At least once a week	9	7.9
At least once a month	29	25.6
At least once a year	43	38.1
Ceased currently*	29	25.6

*Adolescents who have taken none of the above substances in the past three months

### Knowledge of adolescents on SRH issues

The level of knowledge of adolescents abiut SRH services was assessed by using 12 questions. Accordingly, more than half (53.2%) of respondents were knowledgeable (i.e. above the mean score of 6.67) about SRH issues. The majority (66.2%) of respondents had information on SRH services and the school environment was the commonest source of information (85.1%). Nearly three-quarters (72.1%) of adolescents knew at least one method of contraception (Table 3).

**Table 3:** Level of SRH knowledge among rural adolescents of Guraghe zone, Southern Ethiopia, May 1–30, 2020 (n = 1009)

Knowledge assessment variables	Frequency	%
Ever heard about SRH		
Yes	668	66.2
No	341	33.8
Source of information(n = 668)		
From school	568	85.1
Radio	385	57.6
Television	101	15.1
Social media	113	16.8
Family members	242	36.2
Can mention at least one SRH service		
Yes	547	54.2
No	462	45.8
Know delivery points for SRH services		
Yes	443	43.9
No	566	56.1
Know SRH service provider		
Yes	508	50.3
No	501	49.7
Know the reasons for unintended pregnancy		
Yes	583	57.8
No	426	42.2
Know at least one way of avoiding pregnancy		
Yes	532	52.7
No	477	47.3
Know at least one type of STI		
Yes	569	56.4
No	440	43.6
Know the mode of transmission of STI		
Yes	514	50.9
No	495	49.1
Can mention at least one mechanism of STI prevention		
Yes	523	51.8
No	486	48.2
Know the place where STI case management are availed		
Yes	368	36.5
No	641	63.5
Know about the benefits of contraceptive methods		
Yes	787	78.0
No	222	22.0
Know at least one type of contraceptive		
Yes	727	72.1
No	282	27.9
Knowledge by method mix		
Condom	623	85.7
Oral contraceptives	563	77.4
Injectables	384	52.8
Implants	220	30.2
IUD	141	19.3
Overall knowledge		
Knowledgeable	537	53.2
Not knowledgeable	472	46.8

### Sexual and reproductive health service use among adolescents

**Fig. 1:**
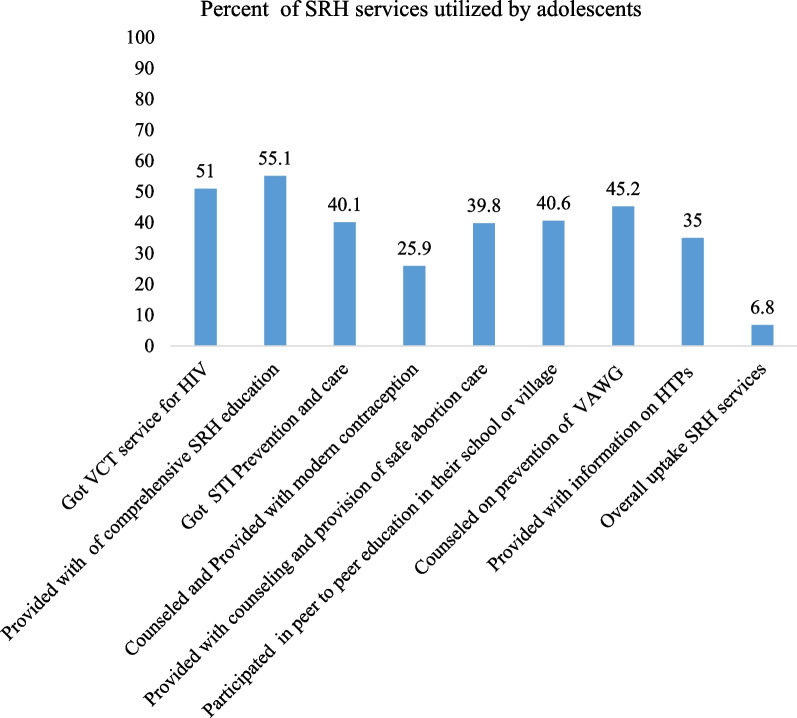
Shows the percentages of SRH services utilized by adolescents in rural districts of Guraghe zone, Southern Ethiopia, 2020

 The use of the SRH service was assessed using eight elements, and the mean (± SD) score of service uptake was 4.05(± 1.94), with only 69(6.8%) of adolescents receiving all key elements. Taking into account the different components of care, the delivery of comprehensive sexuality education (CSE) was the commonest received item by adolescents (55.1%), followed by the VCT service (51.0%). While relatively few respondents (25.9%) were provided with contraceptive information and services (Fig. 1).

### Reasons perceived as barriers to using the SRH service among adolescents

**Fig. 2:**
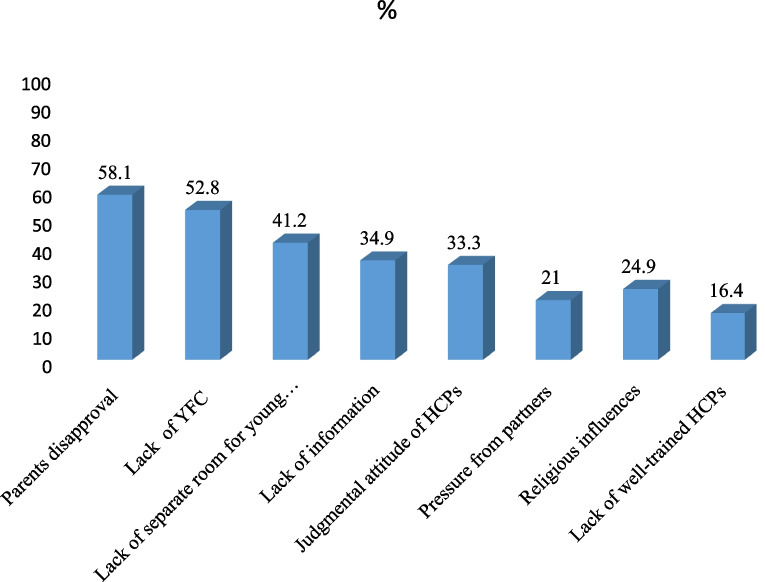
Perceived reasons reported by adolescents as a hindrance to SRH service utilization in rural districts of Guraghe zone, Southern Ethiopia, 2020

Respondents mentioned a lack of skilled healthcare providers, the high cost of facilities and goods and services, the lack of separate youth rooms, and healthcare providers' judgment as barriers to using SRH services (Fig. 2).

### Determinants of SRH service utilization

The variables identified as significant determinants of the use of SRH services in the multivariable generalized linear regression analysis with the Poisson link were: the educational level of adolescents, parental discussion, the availability of youth clubs, and knowledge of SRH-related issues. Adolescents with a secondary level of education had a 28% higher chance of receiving key elements of SRH services as compared to adolescents with no formal education. [AOR:1.28, 95% CI:1.03–1.56]. In their neighboring environment, the availability of a youth club influences SRH usage. Compared to respondents who reported that there was a functioning youth club in their community, adolescents who lacked it were 29% less likely to use SRH services [AOR: 0.71, 95% CI: 0.66–0.87]. The uptake of the SRH service was found to be positively influenced by parent discussions of SRH issues. When compared to their counterparts, adolescents who had parental discussions had 1.31 times the odds of using key elements of SRH services [AOR: 1.31, 95% CI: 1.13–1.51]. Adolescents who were not knowledgeable of SRH issues were 21% less likely to use services compared to those who were knowledgeable [AOR: 0.79, 95% CI: 0.73–0.85)] (Table 4).

**Table 4:** Results of the Multi-variable Generalized Linear regression Analysis with Poisson log link to identify the determinants of SRH service use among adolescents in Guraghe Zone, Southern Ethiopia, May1-30, 2020

Respondents’ characteristics	Mean of number of SRH services	p-value	Beta	AOR(95% CI)
Age				
15–16	3.8	0.000^b^	1	1
17–19	4.4		0.04	1.04(0.98,1.11)
Sex				
Female	4.1	0.546^b^		
Male	4.0			
Marital status				
Un married	4.0	0.410^b^		
Ever Married	3.8			
Current enrolment at school				
No	3.07	0.000^b^		1
Yes	4.14		0.114	1.12(0.98,1.28)
Educational status				
No formal education	3.1	0.000^a^	1	1
Primary	3.5		0.048	1.04(0.91,1.21)
Secondary	4.6		0.173	1.28(1.03,1.56)
Current living arrangement				
With husband/ wife	3.5	0.002^a^		1
With father only	3.1		− 0.157	0.85(0.53,1.36)
With mother only	3.7		0.145	1.15(0.67,1.98)
With both parents	4.1		0.009	1.01(0.79,1.27)
Mother’s education level (n = 969)				
No Formal education	4.0	0.987^a^		
Primary	4.0			
Secondary	4.0			
Diploma and above	3.9			
Father’s educational level				
No formal education	3.9	0.001^a^		1
Primary	3.9		− 0.006	0.99(0.92,1.07)
Secondary	4.4		0.033	1.03(0.94,1.13)
Diploma and above	4.8		0.070	1.07(0.94,1.22)
Family size				
> 5	4.0	0.764^b^		
≤ 5	4.1			
Availability of Youth clubs nearby				
Yes	5.2	0.000^b^		1
No	3.2		− 0.333	0.71(0.66,0.87)
Ever had sexual partner/s				
No	4.1	0.363^b^		
Yes	4.0			
Ever had a parental discussion on SRH issues				
No	3.3	0.000		1
Yes	4.9		0.194	1.31(1.13,1.51)
Overall knowledge				
Knowledgeable	4.8	0.000		1
Not knowledgeable	3.1		− 0.234	0.79(0.73,0.85)

Key: 1: reference category; *AOR* Adjusted odds ratio, p-values with ^a^indicates descriptive analysis by using ONE WAY ANOVA, and p-value with ^b^indicates independent t-test analysis

## Discussion

The Sexual and Reproductive Health(SRH) program for adolescents is one of the critical components of health indicators for immediate and long-term SRH needs [7, 12]. This study sought to examine the uptake of key elements of SHR services by adolescents in rural settings. The present study showed that the average SRH service utilization score was 4.05 with a standard deviation of 1.94 and 6.8% overall utilization. This would suggest that the majority of adolescents in the study area did not have the WHO-recommended content of SRH services. Many studies have reported the low utilization of SRH services among adolescents [19, 20, 22, 33].

In terms of individual service components, 51.0% of youth received VCT services, which is lower than similar studies elsewhere in Ethiopia [26, 27]. The use of family planning was assessed by asking for at least one type of family planning method over their lifetime, and 25.9% of adolescents have used family planning. This is lower than the Mini-EDHS report of 2019 (36.5%) and studies in Ghana (49%), Gondar (79.5%), Gobba (71.4%), Mekele (85.8%), and Anchar district (39.3%) [18, 23, 26, 27, 34, 35] and consistent with a single study among rural adolescents in Awabel district (25.4%) [20]. This may be due to differences in the study setting in which the current study was conducted among rural teens, where it may be difficult for the majority of respondents to find information education communication (IEC) about SRH Key Services, and the inaccessibility of SRH service delivery points, potentially leading to poor service compliance. This rationalization has been well supported by the current study, in which an important segment (44.9%) of adolescents was not able to obtain a comprehensive sexuality education, and several studies elsewhere have also shown low use among adolescents in rural areas [22, 23, 36–38]. Another possible reason for this variance could be the difference between study participants in which the present study consisted of adolescents with sexual experience and inexperienced adolescents, whereas higher prevalence studies focused strictly on adolescents with sexual experience [26, 27].

In addition, this research found that on first sexual contact, the use of contraceptives was only 61.7%. This has shown how frequently these adolescents are exposed to multiple sexual and reproductive health problems, such as unwanted pregnancies, unsafe abortions, STIs, and HIV/AIDS. There is also evidence that the content of all other adolescent SRG services is low, implying that stakeholders need a concerted effort to improve these services.

The level of education of adolescents was found to influence the use of SRH services. This finding was in tandem with some studies conducted elsewhere [22, 26, 38]. This may be because more educated adolescents had a higher chance of learning about those service packages and their benefits, and are more open to new knowledge associated with SRH services with good information-processing skills, which could lead to service usage. This might be well supported by the present study in which adolescents in secondary education were included in about three-quarters (73.5%) of adolescents with a score above the average.

The current study states that discussing with parents has a positive impact on the uptake of SRH services. Some studies carried out in Africa complement this observation [20, 21, 26, 35, 39]. Perhaps this is because, without limitation, youth who were discussing SRH issues with their parents would have gained more information and experience about SRH services and would have been allowed them to use services.

This study found that for adolescents who are not knowledgeable about SRH-related issues, the likelihood of using SRH services is lower than for adolescents who are knowledgeable about SRH-related issues. This is supported by a similar study done in northern Ethiopia [22]. This is plausible because the more youth have adequate knowledge of SRH services, such as their benefits, content, and areas of service delivery, the more they comply with recommended SRH services. Therefore, stakeholders require a collaborative effort to improve adolescents' knowledge of SRH services to finally increase the uptake of services through behavioral change communication interventions.

Adolescents who had no youth club were 29% less likely to use SRH services than adolescents who reported that there was a youth club in their area. This is consistent with research from African nations [30–32]. The possible reason for this could be that as youth clubs become accessible to teens, they could improve the adoption of SRH services by increasing peer-to-peer dialogue so that young clients feel safer and more confident in seeking services [30, 40, 41]. Therefore, the collaborative effort of the agencies involved in expanding these youth clubs to inaccessible districts has proven to be an applicable strategy for the delivery of HRS services to youth.

There are strengths as well as limitations in this research. The study is the first of its kind to comprehensively assess the determinants of SRH practice in this area of study and at the country level, taking into consideration all aspects of care. Although efforts have been made to minimize potential gaps in this report, readers should be cautious in their interpretation of the findings. Given that it had some sensitive issues and focused on self-reporting, respondents may have been exposed to social desirability biases and may have contributed to the under-reporting of some SRH services. Finally, there may be a risk of recall bias because adolescents were interviewed about some events that had already occurred before this study.

## Conclusions

In the study area, the mean score and overall SRH service utilization were low. Educational level, having a parental discussion, lack of youth clubs, and knowledge of SRH issues were identified as significant predictors of the uptake of key elements of SRH services. Schools should be an excellent means of educating adolescents to increase their knowledge of key elements of SRH services. Furthermore, stakeholders must work together to improve the culture of parental discussion with adolescents and establish and strengthen youth clubs, as measures for encouraging the use of SRH services.

## Data Availability

The data used to strengthen the results of this study are to be had from the corresponding author based on reasonable request via the email address akliluhabte57@gmail.com.

## Abbreviations

AOR: Adjusted odds ratio
CSE: Comprehensive sexuality education
EDHS: Ethiopian demographic health survey
FMOH: Federal ministry of health
HTP: Harmful traditional practices
SNNPR: Southern nations nationality, and people region
SRH: Sexual and reproductive health
STI: Sexually transmitted infection
VAWG: Violence against women and girls
VCT: Voluntary HIV/AIDS counseling, and testing
WHO: World Health Organization
